# The cortical vestibular system: insights from electroencephalography

**DOI:** 10.1097/WCO.0000000000001448

**Published:** 2025-12-02

**Authors:** Adolfo M. Bronstein, Jasmine L. Mirdamadi, Toby J. Ellmers

**Affiliations:** a Department of Brain Sciences, Imperial College London, UK; b Department of Rehabilitation Medicine, Emory University, Atlanta, Georgia; c Department of Rehabilitation and Movement Science, University of Vermont, Burlington, Vermont, USA

**Keywords:** balance, cortical processing, electroencephalography, self-motion, vestibular perception

## Abstract

**Purpose of review:**

Although electroencephalography (EEG) is central to epilepsy diagnosis, its role in patients presenting with dizziness or balance disorders has historically been negligible. This review provides a timely synthesis of recent methodological and conceptual advances demonstrating how modern EEG analyses can probe cortical contributions to vestibular and balance function.

**Recent findings:**

While vestibular epilepsy remains rare, EEG is increasingly being applied to investigate cortical dynamics during vestibular stimulation, postural control, and balance perturbations. Contemporary analytic techniques have revealed that alpha-band and beta-band EEG activity reflect key aspects of vestibular perception, adaptation, and postural control. Findings in patients with higher order vestibular dysfunction link symptoms to abnormal oscillatory patterns corresponding to disrupted sensory integration and maladaptive attentional engagement. Advances in mobile EEG approaches now permit reliable signal acquisition during movement and direct vestibular stimulation, allowing quantification of ecologically relevant cortical responses such as the perturbation-evoked potential.

**Summary:**

EEG provides a powerful, accessible, and scalable tool to characterize cortical contributions to vestibular processing and balance. These developments highlight its emerging value for identifying neurophysiological biomarkers of vestibular dysfunction, improving diagnostic precision, and informing targeted rehabilitation strategies.

## INTRODUCTION

Although electroencephalography (EEG) is integral to the routine diagnosis and management of epilepsy, it has historically held limited clinical utility for patients presenting with dizziness or balance disorders in outpatient settings. This, therefore, marks the first occasion on which the Neuro-otology section of *Current Opinion in Neurology* features a dedicated discussion of EEG. Two clarifications are warranted. First, although this review focuses on recent advances rather than providing an exhaustive historical account, relevant earlier publications are also included given the lack of prior coverage in this area. Second, as EEG currently offers little direct diagnostic value for the dizzy patient, much of the work discussed will be research-focused, particularly on how contemporary EEG analytic approaches are enhancing our understanding of balance control and its disorders (see section ‘Modern research applications of EEG within neuro-otology'). Preceding this, a brief overview will address the single neuro-otological condition for which EEG remains essential to diagnosis: vestibular epilepsy (or vertiginous seizures). 

**Box 1:**
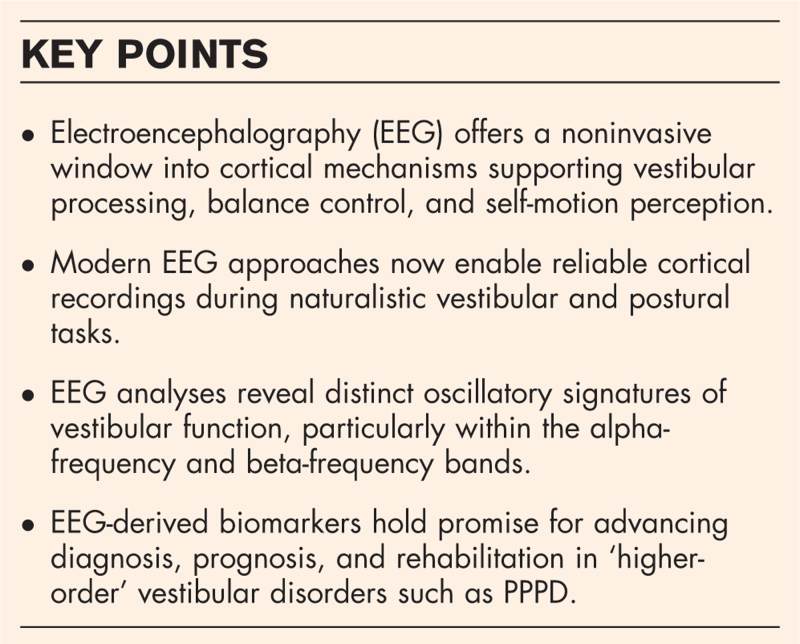
no caption available

## CLINICAL APPLICATIONS OF ELECTROENCEPHALOGRAPHY IN NEURO-OTOLOGY: VESTIBULAR EPILEPSY

Vertigo of epileptic origin is exceptionally rare. By way of disclosure, the first author (A.M.B.) has yet to encounter a convincing case in over 50 years of clinical neurology and neuro-otology practice. In most situations, paroxysmal vertigo attributed to ‘vestibular epilepsy’ is ultimately explained by more common peripheral causes such as benign paroxysmal positional vertigo (BPPV), presyncopal dizziness, or vestibular paroxysmia. Nonetheless, there are no neurophysiological grounds to dismiss the possibility outright. As shown by Penfield's classic intraoperative studies [1], electrical stimulation of the temporal cortex can evoke vivid vestibular sensations, including vertigo.

A recent case report described EEG abnormalities occurring synchronously with vertiginous symptoms on routine recordings, further confirmed by sphenoidal electrodes showing paroxysmal activity in mesial and mid-temporal regions [2]. The authors provided a thorough list of differential diagnoses (including BPPV, Ménière's disease, transient ischaemic attack, vertebrobasilar insufficiency, basilar migraine, and others), but notably omitted vestibular paroxysmia. This omission is notable, as vestibular paroxysmia represents a well established disorder of the vestibular nerve characterized by frequent, brief paroxysms of vertigo, oscillopsia and lateropulsion. Vestibular paroxysmia can be caused by neurovascular cross-compression in the cerebellopontine angle where high-resolution MRI with 3D-CISS or FIESTA sequences remains the preferred imaging modality. Beware that such vascular contacts are also found in approximately half of healthy controls [3^▪^].

A recent case-based book described two patients with vestibular paroxysmia and one with vestibular epilepsy, the former including paroxysmal ‘electric’ crackling noises in one ear–a phenomenon that helped localize the disorder [4]. The omission by Tsai *et al.*[2] to consider vestibular paroxysmia as a differential diagnosis of an otherwise convincing case of vestibular epilepsy reminded us of informal conversations with epileptologists who on occasions can sound extremely sceptical of vestibular paroxysmia. To some extent, such scepticism seems to have re-emerged in a new case series of patients with vestibular epilepsy [5^▪^]. Indeed, the abstract states: ‘… diagnostic criteria (for epileptic vertigo) overlap with the criteria for vestibular paroxysmia, suggesting the possibility of a single nosological entity’. This case series reports 31 patients with vestibular epilepsy (out of a database of 2000 patients from a single epilepsy centre). Although 10 patients presented with vertigo or disequilibrium as isolated symptoms, classic epileptic features such as loss of awareness (71%), falls (68%), and tonic–clonic seizures (39%) were common. These findings accord with the comprehensive review by Tarnutzer *et al.*[6], which concluded that epileptic vertigo is uncommon, and isolated vertiginous seizures are exceptionally rare. Foci tend to be more frequently temporal than parietal and are distributed equally across hemispheres. Ultimately, the scarcity of well documented cases (only four with concurrent ictal EEG, MRI, and full vestibular testing in Tarnutzer's review) highlights the limitations of current evidence. Progress in understanding this rare condition will likely depend on prospective, multicentre collaborations linking epilepsy and neuro-otology services.

## MODERN RESEARCH APPLICATIONS OF ELECRTOENCEPHALOGRAPHY WITHIN NEURO-OTOLOGY: FROM VESTIBULAR-EVOKED POTENTIALS TO MARKERS OF SELF-MOTION PERCEPTION

### Elecrtoencephalography: a brief primer

EEG provides a noninvasive, cost-effective means of recording neural activity with millisecond temporal precision. Unlike other neuroimaging modalities such as functional MRI (fMRI) or PET, which offer superior spatial localization but are limited by slow hemodynamic signals, EEG provides a direct and real-time measure of neural activity that is widely used in clinical and research settings. In the context of dizziness and vestibular disorders, EEG provides a valuable means of exploring the cortical mechanisms that support self-motion processing and perception, spatial orientation, and balance regulation during real-world tasks likely to induce dizziness or imbalance. It also enables the identification of maladaptive oscillatory or connectivity patterns that may contribute to higher-order/subjective dizziness and disequilibrium.

EEG primarily reflects the summed postsynaptic potentials of synchronized populations of cortical pyramidal neurons, thereby providing a real-time index of excitatory and inhibitory processes within superficial cortical layers [7,8]. This high temporal resolution (as well as the ability to measure signals whilst the participant is mobile) makes EEG particularly valuable for studying transient neural events that underlie sensory integration, attention, and motor control (including balance). Decomposing EEG signal into specific frequency bands (e.g. 8–12 Hz alpha band or 15–30 Hz beta band activity) can also provide specific insight into underlying neurobiological mechanisms [8]. For instance, alpha appears to negatively correlate with cortical activation, in that *reduced* alpha band activity reflects selective cortical processing of a given stimulus [9].

Despite its advantages, EEG faces several challenges, particularly the contamination of cortical signals by extra-cranial artifacts such as eye blinks and muscle activity. This issue is especially pronounced when studying vestibular function, as vestibular stimulation naturally elicits robust eye movements. However, as described in detail below, recent advances in signal processing and artifact correction have now made it possible to obtain reliable EEG recordings during vestibular and postural protocols. These methodological developments open new opportunities to examine cortical contributions to vestibular processing, extending investigation beyond the brainstem reflexes that have traditionally been the focus of clinical vestibular testing.

### Assessing electroencephalography during vestibular stimulation

Some of the earliest research employing EEG to vestibular processing described a ‘vestibular evoked potential’ [10]: a slow-rising, negative cortical potential occurring at central electrodes in response to (Barany chair) rotational stimuli. However, recent studies using more advanced signal processing techniques have raised questions about the vestibular specificity of this evoked response, as similar potentials are observed in individuals with and without vestibular loss following artifact removal (e.g. eye and muscle) [11]. This contrasts with analytical approaches that have explored (postartifact removal) oscillatory responses to vestibular stimuli at different frequencies: here, clear differences are observed between controls and patients with bilateral vestibular loss [11]. Whilst healthy controls exhibited robust bilateral EEG alpha (8–12 Hz) suppression across temporo-parietal scalp regions in response to yaw-plane rotations, this activity is markedly reduced in vestibular loss patients. Given current understanding about the *inhibitory* role of alpha activation [9], the authors proposed ‘…suppression of oscillations in the alpha band over temporo-parietal scalp regions reflects cortical vestibular processing, potentially comparable with alpha and mu oscillations in the visual and sensorimotor systems’ (p. 1228). Interestingly, recent research highlights broader alpha-band disturbances in patients with bilateral vestibular loss, showing reduced EEG ‘alpha reactivity’ in visual cortical areas upon eye closure [12^▪^]. These findings suggest that vestibular loss may disrupt sensory-related alpha dynamics more generally, through cross-modal mechanisms. Of potential clinical relevance, it has also been shown that vestibular adaptation (critical to overcoming vertigo), as elicited by asymmetric rotation on a Barany chair, is reflected in alpha rhythm changes in frontoparietal attentional networks [13]. This raises the possibility that the absence of such signal could be a potential biomarker of disrupted central adaptation following an acute vestibular insult (e.g. as often occurs in persistent postural-perceptual dizziness (PPPD)).

EEG is also increasingly being incorporated into existing clinical vestibular tests. For example, Romero *et al*. [14] recently characterized cortical EEG responses during caloric vestibular stimulation, demonstrating the feasibility of capturing vestibular-evoked cortical activity during clinical assessments. They reported widespread alpha-band (8–12 Hz) suppression that was not correlated with caloric-induced slow-phase velocity, whereas perceived self-motion intensity showed a strong positive association with beta-band (13–30 Hz) activation (*r* = 0.775, *P* = 0.008). These findings may have important implications for ‘higher-order’ vestibular disorders, which are thought to involve disturbances in perceptual rather than reflex-level vestibular processing, including PPPD, ‘unexplained dizziness’ in older adults, and vestibular agnosia (absence/reduction of vestibular perception despite intact peripheral function [15]). Building on these advances, Duncan *et al.*[16,17] recently demonstrated that sophisticated artifact-rejection methods can enable reliable EEG recordings during electrical vestibular stimulation (e.g. Galvanic vestibular stimulation), a context in which substantial signal distortion has historically precluded meaningful analysis. This opens the possibility of assessing higher order vestibular processing in dizzy patients during ecologically relevant motor tasks in which symptoms typically occur, such as posture, gait or spatial navigation.

### Electroencephalography and balance: evidence from quiet stance

EEG can also reveal changes in cortical engagement as a function of postural task difficulty. Edwards *et al.*[18] demonstrated that the difficulty of a postural balance task is reflected in the suppression of EEG alpha rhythm (8–12 Hz). Specifically, more challenging tasks produced greater reductions in alpha power in bilateral central and parietal cortices, with the right hemisphere showing more pronounced changes. This is presumably a measure of the additional cortical resources recruited to meet task demands.

Applying similar methods to patient populations, Ibitoye *et al.*[19] examined sitting-to-standing EEG changes in older adults with ‘unexplained dizziness’, a group where diagnosis remains difficult (i.e. not due to vestibular disorders or orthostatic hypotension). Interestingly, older adults without dizziness exhibited enhanced EEG suppression (central theta (4–7 Hz) and alpha, as above) when standing compared with younger controls. Individuals with unexplained dizziness showed even greater suppression relative to age-matched healthy controls. This finding suggests that the brains of elderly dizzy patients are biologically ‘older’ than those of nondizzy peers. In support of this claim, prospective studies [20,21] further indicate that disruption of postural EEG networks in these patients is at least partly attributable to small vessel disease and microstructural white matter changes, as evidenced by fractional anisotropy alterations on MRI (for a recent review in this journal, see [22]).

## USING ELECTOENCEPHALOGRAPHY TO QUANTIFY POSTURAL INSTABILITY

### Perturbation-evoked potentials

**FIGURE 1:**
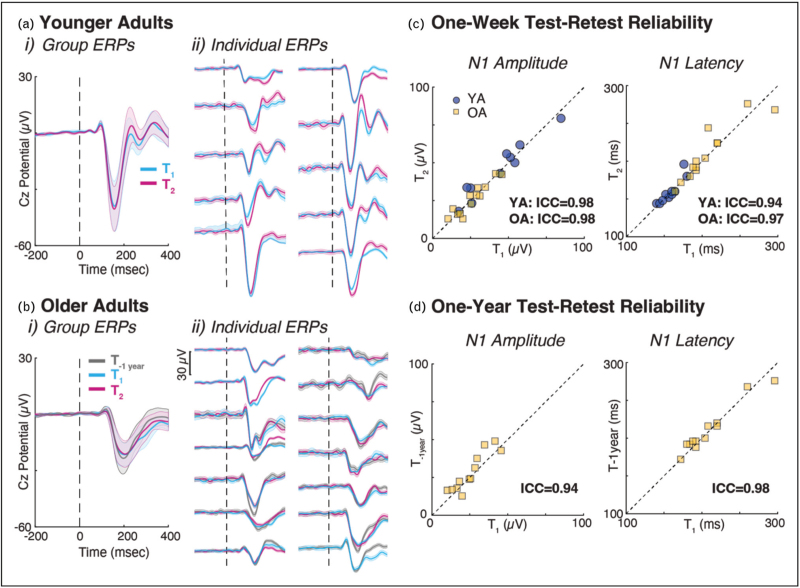
Test-retest reliability across sessions. Perturbation-evoked potentials for young adults (YA; a) and older adults (OA; b) at the group level (i) and individual level (ii). T_1_ and T_2_ separated by ~1 week. T_1_ and T_-1_ _year_ separated by ~1 year. Solid line represents the mean derived from all trials, and shaded region represents the standard deviation. Vertical dashed line denotes perturbation onset. Test–retest reliability for N1 amplitude (left column) and latency (right column) across a 1-week (c) and 1-year time span (d). Dashed diagonal denotes line of equality. Figure republished from Ref. [32^▪▪^], CC BY 4.0 (http://creativecommons.org/licenses/by/4.0/).

Advance in mobile brain/body imaging (MoBI) provides a unique opportunity to characterize cortical mechanisms underlying dizziness and vestibular dysfunction in more ecologically relevant paradigms than quiet standing. A sudden loss of balance elicits a perturbation-evoked cortical potential thought to reflect cognitive and sensorimotor processing involved in balance. The most well characterized signal is the N1, a large negative voltage deflection that peaks 100–200 ms after a balance disturbance [23,24]. The N1 is modulated by several factors including perturbation magnitude [25,26], perturbation predictability [27,28], attention [29,30], and perceived threat [31], and may differentiate between groups that commonly have balance impairments. For instance, older adults have smaller and slower responses compared to younger adults [32^▪▪^], and stroke survivors have smaller and slower responses compared to age-matched controls [33]. Even in the absence of group differences, individual differences in N1 responses are correlated to balance [34,35] and cognitive function [36], highlighting its clinical potential. These cortical signatures not only stratify individuals but also have excellent test–retest reliability up to 1 year in younger and older adults (see Fig. 1[32^▪▪^]), a necessary precursor towards biomarker validation. Clinically, the N1 can be reliably measured with only three electrodes, six trials, and simple, automated signal processing, with nearly identical outcomes to that obtained with high-density EEG that is time-intensive and requires expertise both in the setup and signal processing [32^▪▪^]. The stability and simplicity of the N1 supports its usage in longitudinal studies to objectively quantify subtle functional declines not captured by standard behavioural assessments, enabling earlier detection and treatment. The N1 can also be reliably elicited using a low-cost tethered lean-and-release system [37] that parallels manual clinical tests (e.g. miniBEST), providing a practical and precise neurophysiological index of postural control that in the future could enhance individual stratification and clinical decision-making.

Cortical activity during balance control is also sensitive to experimental manipulations of attention and expectation, offering insight into maladaptive cognitive–perceptual mechanisms in individuals with PPPD and unexplained dizziness in older adults. For instance, Parr *et al.*[30] have demonstrated that directing hypervigilant attention toward balance – an attentional style characteristic of both PPPD and unexplained dizziness in older adults [38] – reduces the amplitude of the N1 and leads to greater postural instability. These results suggest that excessive top-down attentional engagement may disrupt the integration of sensory feedback required for stable postural control [39^▪^]. Relatedly, our recent work [40] demonstrates that aberrant expectations of postural instability during perturbations can induce subjective perceptions of imbalance in healthy controls similar to those observed in PPPD [41], with these effects seemingly driven by anticipatory (i.e. preperturbation) disturbances in EEG. Collectively, these findings point to identifiable neurophysiological markers of maladaptive cognitive–perceptual processes during balance. Combined with low-cost, accessible approaches such as the lean-and-release test and a single EEG electrode at Cz (see above), these markers could provide actionable targets for both assessment and treatment in patients with ‘higher order’ or functional vestibular disorders.

### Naturally occurring postural sway: using electroencephalography to better quantify quiet stance

**FIGURE 2:**
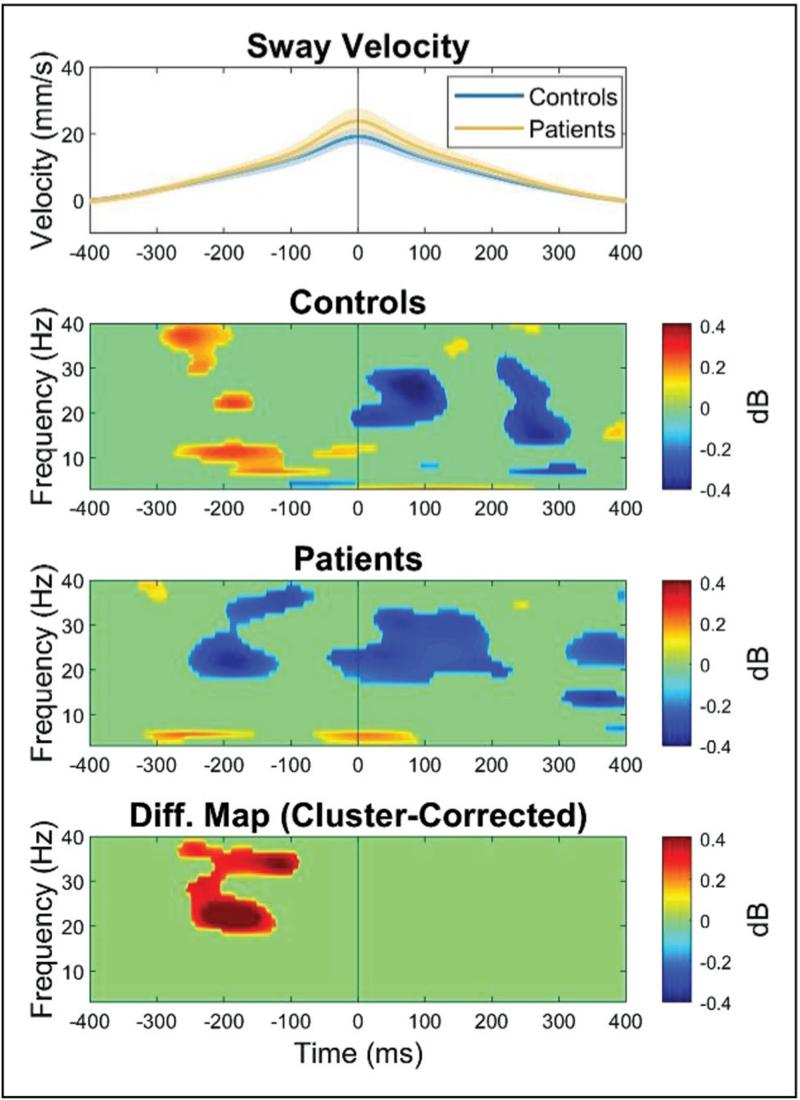
Grand-averaged sway velocity (top panel) and EEG time–frequency plots for older adult controls (second panel) and older patients with ‘unexplained dizziness’ (third panel) at electrode Cz, time-locked to the peak of micro-fall event (timepoint-0). Statistically significant increases in power relative to baseline are displayed in red and significant decreases in power relative to baseline are in blue (*P* < 0.05, permutation-based statistics). The bottom panel presents the statistically significant between-group differences (cluster-corrected to *P* < 0.05). As can be seen, Controls exhibited an increase in high frequency (high beta and low gamma) EEG activity between 250 and 150 ms prior to peak instability, whist patients show a marked suppression in these same EEG frequency bands. Figure republished from Ref. [39^▪^], CC BY 4.0 (http://creativecommons.org/licenses/by/4.0/).

Recent work indicates that event-related EEG responses can extend beyond externally induced balance perturbations and provide valuable insight into the ongoing regulation of posture and balance. Even during quiet standing, balance is characterized by frequent (~every 5 s) fall-like events, termed ‘micro-falls’ (see top panel of Fig. 2), which arise from the natural forward positioning of the body's centre of mass. Consequently, averaging EEG activity across an entire trial of quiet stance may obscure important neural dynamics, as the signal encompasses distinct phases of falling, recovery, and relative equilibrium. Nakamura *et al.*[42] recently reported that the regulation of these micro-falls is associated with distinct EEG patterns: beta activation across electrode Cz during the early phase of the micro-fall, followed by beta suppression prior to the recovery phase (i.e. the arrest and reversal of the fall). Given the established role of cortical beta activity in sensory and motor inhibition [43], the authors interpreted these temporally specific beta-band responses as reflecting a cortical mechanism that interrupts the micro-fall and monitors re-afferent sensory feedback. Yet, these micro-falls typically take place outside of conscious awareness [44]. As patients with Phobic Postural Vertigo (a predecessor of PPPD) seem to become aware of this incessant to-and-fro movements inherent to normal body sway [45] applying this analysis technique would be highly relevant to PPPD patients. Assessing cortical and perceptual responses to micro-falls may, therefore, provide novel insight into vestibular and dizziness disorders characterized by distorted perceptions of imbalance, such as PPPD and unexplained dizziness in older adults.

Building on this work, Ellmers *et al.*[39^▪^] recently applied this analytical approach to older adults with unexplained dizziness and matched controls. They reanalysed EEG data recorded during quiet standing [19], segmenting the signal into epochs time-locked to micro-fall events (defined as peaks in sway velocity exceeding 3 SDs of the mean). Interestingly, patients were not objectively more unstable than controls: neither the number nor the magnitude of instability bouts differed significantly between groups (see Fig. 2, top panel). However, patients reported markedly greater subjective unsteadiness (*P* < 0.001), consistent either with a perceptual imbalance or with an over-cautious protective stepping response [21]. EEG analyses (focused on electrode Cz) revealed distinct cortical signatures: whereas controls exhibited the characteristic beta activation–suppression sequence described by Nakamura *et al.*[42], patients showed sustained beta suppression throughout the micro-fall (see Fig. 2, bottom panels). Based on prior findings that reduced preperturbation beta power is associated with enhanced perceptual sensitivity in neurotypical younger adults [28,46], reduced beta power in older adults with dizziness may reflect enhanced sensitivity to aberrant sensory signals that leads to subjective postural instability. Clinically, this heightened sensitivity may cause normally imperceptible micro-falls to enter conscious awareness and manifest as perceived dizziness, representing a potential mechanism underlying balance disorders characterized by subjective or distorted perceptions of instability.

## CONCLUSION

Together, these advances position EEG as a powerful tool for bridging basic vestibular neuroscience and clinical practice. By enabling direct measurement of cortical contributions to vestibular processing, balance, and self-motion, EEG holds promise for improving the assessment and treatment of complex dizziness and balance disorders. Future research should now focus on translating these mechanistic insights into clinical neuro-otology. One particularly promising avenue is EEG-based neurofeedback, which provides real-time feedback of cortical activity that individuals can learn to modulate through trial and error [47]. Although not yet widely applied to vestibular disorders, this approach could offer a novel means of targeting maladaptive cortical activity and perceptual disturbances characteristic of higher order vestibular syndromes.

## Acknowledgements

*None*.

### Financial support and sponsorship

*This work was supported by a Wellcome Trust Sir Henry Wellcome Fellowship awarded to T.J.E. (Grant Number: 222747/Z/21/Z).*

### Conflicts of interest

*There are no conflicts of interest.*

## REFERENCES AND RECOMMENDED READING

Papers of particular interest, published within the annual period of review, have been highlighted as:

▪ of special interest
▪▪ of outstanding interest

## References

[1] Penfield W. Vestibular sensation and the cerebral cortex. Ann Otol Rhinol Laryngol 1957; 66:691–698. 13488343 10.1177/000348945706600307

[2] Tsai CH Chen TS Lai MC Huang CW. Unilateral nonlesional temporal lobe epilepsy presenting as isolated ictal vertigo: a case report. J Int Med Res 2023; 51:3000605231187801. 37548224 10.1177/03000605231187801 PMC10408343

[3▪] Dieterich M Brandt T. Vestibular paroxysmia: a systematic review. J Neurol 2025; 272:188. 39932594 10.1007/s00415-025-12913-8 PMC11814022

[4] Bronstein AM Kaski D. Puzzling cases in vertigo & dizziness. 2023; Tree Life Media (a division of Kothari Medical), 164.

[5▪] Perriguey M Elziere M Lopez C Bartolomei F. Vestibular epilepsy: clinical and electroencephalographic characteristics with the proposed diagnostic criteria. J Neurol 2024; 272:68. 39680238 10.1007/s00415-024-12796-1

[6] Tarnutzer AA Lee SH Robinson KA. Clinical and electrographic findings in epileptic vertigo and dizziness: a systematic review. Neurology 2015; 84:1595–1604. 25795644 10.1212/WNL.0000000000001474 PMC4408281

[7] Cohen MX. Where does EEG come from and what does it mean? Trends Neurosci 2017; 40:208–218. 28314445 10.1016/j.tins.2017.02.004

[8] Hari R Puce A. MEG-EEG primer. 2nd ed New York, NY: Oxford University Press; 2023.

[9] Klimesch W Sauseng P Hanslmayr S. EEG alpha oscillations: the inhibition–timing hypothesis. Brain Res Rev 2007; 53:63–88. 16887192 10.1016/j.brainresrev.2006.06.003

[10] Hood JD Kayan A. Observations upon the evoked responses to natural vestibular stimulation. Electroencephalogr Clin Neurophysiol 1985; 62:266–276. 2408873 10.1016/0168-5597(85)90004-8

[11] Gale S Prsa M Schurger A. Oscillatory neural responses evoked by natural vestibular stimuli in humans. J Neurophysiol 2016; 115:1228–1242. 26683063 10.1152/jn.00153.2015 PMC4808125

[12▪] Ibitoye RT Castro P Ellmers TJ. Vestibular loss disrupts visual reactivity in the alpha EEG rhythm. NeuroImage Clin 2023; 39:103469. 37459699 10.1016/j.nicl.2023.103469 PMC10368920

[13] Cooke JI Guven O Castro Abarca P. Electroencephalographic response to transient adaptation of vestibular perception. J Physiol 2022; 600:3517–3535. 35713975 10.1113/JP282470 PMC9544486

[14] Romero DJ Chang C Clay D. Exploratory analysis of cortical-vestibular interaction: the relation between caloric-induced changes in electroencephalography frequency bands, the vestibulo-ocular reflex, and perception. Ear Hear 2025. [Online ahead of print]. 10.1097/AUD.0000000000001710 40745445

[15] Calzolari E Chepisheva M Smith RM. Vestibular agnosia in traumatic brain injury and its link to imbalance. Brain J Neurol 2021; 144:128–143. 10.1093/brain/awaa386 PMC7880674 33367536

[16] Duncan SJ Kamyla M Ferguson HJ Wilkinson DT. Extraction of the GVS electrical artifact from EEG recordings of the motor related cortical potential. J Neurosci Methods 2022; 368:109459. 34954254 10.1016/j.jneumeth.2021.109459

[17] Duncan SJ Marques K Fawkes J. Galvanic vestibular stimulation modulates EEG markers of voluntary movement in Parkinson's disease. Neuroscience 2024; 555:178–183. 39074577 10.1016/j.neuroscience.2024.07.048

[18] Edwards AE Guven O Furman MD. Electroencephalographic correlates of continuous postural tasks of increasing difficulty. Neuroscience 2018; 395:35–48. 30391529 10.1016/j.neuroscience.2018.10.040

[19] Ibitoye RT Castro P Desowska A. Small vessel disease disrupts EEG postural brain networks in “unexplained dizziness in the elderly.”. Clin Neurophysiol 2021; 132:2751–2762. 34583117 10.1016/j.clinph.2021.07.027 PMC8559782

[20] Ibitoye RT Castro P Cooke J. A link between frontal white matter integrity and dizziness in cerebral small vessel disease. NeuroImage Clin 2022; 35:103098. 35772195 10.1016/j.nicl.2022.103098 PMC9253455

[21] Castro P Ibitoye R Ellmers T. Towards an explanation for “unexplained” dizziness in older people. Age Ageing 2024; 53:afae137. 38965033 10.1093/ageing/afae137 PMC11223895

[22] Bronstein AM Kattah J. Vascular neuro-otology: vestibular transient ischemic attacks and chronic dizziness in the elderly. Curr Opin Neurol 2024; 37:59–65. 38032270 10.1097/WCO.0000000000001229 PMC10779463

[23] Purohit R Bhatt T. Mobile brain imaging to examine task-related cortical correlates of reactive balance: a systematic review. Brain Sci 2022; 12:1487. 36358413 10.3390/brainsci12111487 PMC9688648

[24] Varghese JP McIlroy RE Barnett-Cowan M. Perturbation-evoked potentials: Significance and application in balance control research. Neurosci Biobehav Rev 2017; 83:267–280. 29107828 10.1016/j.neubiorev.2017.10.022

[25] Solis-Escalante T Stokkermans M Cohen MX Weerdesteyn V. Cortical responses to whole-body balance perturbations index perturbation magnitude and predict reactive stepping behavior. Eur J Neurosci 2021; 54:8120–8138. 32931066 10.1111/ejn.14972 PMC9290492

[26] Payne AM Hajcak G Ting LH. Dissociation of muscle and cortical response scaling to balance perturbation acceleration. J Neurophysiol 2019; 121:867–880. 30517039 10.1152/jn.00237.2018 PMC6520627

[27] Adkin AL Quant S Maki BE McIlroy WE. Cortical responses associated with predictable and unpredictable compensatory balance reactions. Exp. Brain Res 2006; 172:85–93. 10.1007/s00221-005-0310-9 16418848

[28] Mirdamadi JL Ting LH Borich MR. Distinct cortical correlates of perception and motor function in balance control. J Neurosci 2024; 44:e1520232024. 38413231 10.1523/JNEUROSCI.1520-23.2024 PMC11007305

[29] Quant S Adkin AL Staines WR. The effect of a concurrent cognitive task on cortical potentials evoked by unpredictable balance perturbations. BMC Neurosci 2004; 5:18. 15147586 10.1186/1471-2202-5-18 PMC428574

[30] Parr JVV Mills R Kal E. A “Conscious” loss of balance: directing attention to movement can impair the cortical response to postural perturbations. J Neurosci 2024; 44:e0810242024. 39358045 10.1523/JNEUROSCI.0810-24.2024 PMC11604137

[31] Adkin AL Campbell AD Chua R Carpenter MG. The influence of postural threat on the cortical response to unpredictable and predictable postural perturbations. Neurosci Lett 2008; 435:120–125. 18337005 10.1016/j.neulet.2008.02.018

[32▪▪] Mirdamadi JL Poorman A Munter G. Excellent test-retest reliability of perturbation-evoked cortical responses supports feasibility of the balance N1 as a clinical biomarker. J Neurophysiol 2025; 133:987–1001. 39993029 10.1152/jn.00583.2024 PMC12244522

[33] Palmer JA Payne AM Mirdamadi JL. Delayed cortical responses during reactive balance after stroke associated with slower kinetics and clinical balance dysfunction. Neurorehabil Neural Repair 2025; 39:16–30. 39328051 10.1177/15459683241282786 PMC11723813

[34] Payne AM McKay JL Ting LH. The cortical N1 response to balance perturbation is associated with balance and cognitive function in different ways between older adults with and without Parkinson's disease. Cereb Cortex Commun 2022; 3:tgac030. 36043162 10.1093/texcom/tgac030 PMC9415190

[35] Payne AM Ting LH. Worse balance is associated with larger perturbation-evoked cortical responses in healthy young adults. Gait Posture 2020; 80:324–330. 32593102 10.1016/j.gaitpost.2020.06.018 PMC7436194

[36] Payne AM Palmer JA McKay JL Ting LH. Lower cognitive set shifting ability is associated with stiffer balance recovery behavior and larger perturbation-evoked cortical responses in older adults. Front Aging Neurosci 2021; 13:742243. 34938171 10.3389/fnagi.2021.742243 PMC8685437

[37] Payne AM Schmidt NB Meyer A Hajcak G. The balance N1 is larger in anxious children and associated with the error-related negativity. Biol Psychiatry Glob Open Sci 2024; 5:100393. 39526024 10.1016/j.bpsgos.2024.100393 PMC11546193

[38] Ellmers TJ Kal EC. Exploring the role of attention towards balance in chronic dizziness: development of the Balance Vigilance Questionnaire. Eur J Neurol 2024; 31:e16148. 38015469 10.1111/ene.16148 PMC11235928

[39▪] EllmersTJIbitoyeRCastroP. Chronic dizziness in older adults: disrupted sensorimotor EEG beta oscillations during postural instability. Clin Neurophysiol 2025; 174:31–36.40198974 10.1016/j.clinph.2025.03.032

[40] Parr JVV, Mills R, Kal EC, *et al*. Perceiving instability: how expectations bias sensorimotor processing in balance control. bioRxiv 2025. doi: 10.1101/2025.11.18.688816.

[41] San Pedro Murillo E Bancroft MJ Koohi N. Postural misperception: a biomarker for persistent postural perceptual dizziness. J Neurol Neurosurg Psychiatry 2023; 94:165–166. 35995549 10.1136/jnnp-2022-329321

[42] Nakamura A Miura R Suzuki Y. Discrete cortical control during quiet stance revealed by desynchronization and rebound of beta oscillations. Neurosci Lett 2023; 814:137443. 37591357 10.1016/j.neulet.2023.137443

[43] Engel AK Fries P. Beta-band oscillations—signalling the status quo? Curr Opin Neurobiol 2010; 20:156–165. 20359884 10.1016/j.conb.2010.02.015

[44] Fitzpatrick RC Taylor JL McCloskey DI. Ankle stiffness of standing humans in response to imperceptible perturbation: reflex and task-dependent components. J Physiol 1992; 454:533–547. 1474502 10.1113/jphysiol.1992.sp019278 PMC1175619

[45] Qin C Zhang R Yan Z. Research progress on the potential pathogenesis of persistent postural-perceptual dizziness. Brain Behav 2025; 15:e70229. 39740787 10.1002/brb3.70229 PMC11688117

[46] Shin H Law R Tsutsui S. The rate of transient beta frequency events predicts behavior across tasks and species. eLife 2017; 6:e29086. 29106374 10.7554/eLife.29086 PMC5683757

[47] Diotaiuti P Marotta G Vitiello S. Biofeedback for motor and cognitive rehabilitation in parkinson's disease: a comprehensive review of non-invasive interventions. Brain Sci 2025; 15:720. 40722311 10.3390/brainsci15070720 PMC12293922

